# Changes in rumen fermentation and bacterial profiles after administering *Lactiplantibacillus plantarum* as a probiotic

**DOI:** 10.14202/vetworld.2022.1969-1974

**Published:** 2022-08-19

**Authors:** Wulansih Dwi Astuti, Roni Ridwan, Rusli Fidriyanto, Rohmatussolihat Rohmatussolihat, Nurul Fitri Sari, Ki Ageng Sarwono, Ainissya Fitri, Yantyati Widyastuti

**Affiliations:** Research Center for Applied Zoology, National Research and Innovation Agency (BRIN), Cibinong 16911, West Java, Indonesia

**Keywords:** *Lactiplantibacillus plantarum*, microbes, probiotic, rumen

## Abstract

**Background and Aim:**

*Lactiplantibacillus plantarum* is one of the lactic acid bacteria that is often used as probiotics. This study aimed to evaluate the effects of *Lactiplantibacillus plantarum* TSD10 as a probiotic on rumen fermentation and microbial population in Ongole breed cattle.

**Materials and Methods:**

This study adopted an experimental crossover design, using three-fistulated Ongole breed cattle. Treatments were as follows: T0, control without probiotic; T1, 10 mL probiotic/day; T2, 20 mL probiotic/day; and T3, 30 mL probiotic/day. The basal diet of the cattle comprised 70% concentrate: 30% elephant grass (*Pennisetum purpureum*). The concentration of probiotic used was 1.8 × 10^10^ colony-forming unit (CFU)/mL.

**Results:**

We observed significantly lower acetate production compared with control (64.12%), the lowest values being in the T3 group (55.53%). Contrarily, propionate production significantly increased from 18.67% (control) to 23.32% (T2). All treatments yielded significantly lower acetate–propionate ratios than control (3.44), with the lowest ratio in the T3 group (2.41). The protozoal number decreased on probiotic supplementation, with the lowest population recorded in the T2 group (5.65 log cells/mL). The population of specific rumen bacteria was estimated using a quantitative polymerase chain reaction. We found that the population of *L. plantarum, Ruminococcus flavefaciens*, and *Treponema bryantii*, did not change significantly on probiotic supplementation, While that of *Ruminococcus albus* increased significantly from 9.88 log CFU/mL in controls to 12.62 log CFU/mL in the T2 group.

**Conclusion:**

This study showed that the optimum dosage of *L. plantarum* TSD10 as a probiotic was 20 mL/day. The effect of *L. plantarum* as a probiotic on feed degradation in rumen was not evaluated in this experiment. Therefore, the effect of *L. plantarum* as a probiotic on feed degradation should be performed in further studies.

## Introduction

Probiotics are emerging as safe and viable alternatives to antibiotics in livestock rearing [1]. They are non-pathogenic and non-toxic live microorganisms capable of exerting a beneficial effect on the host at the appropriate dosage [2]. Probiotics contribute to the balance of microflora in the digestive tract by increasing the proportion of good microbes [3].

In ruminants, probiotics should have the ability to enhance intestinal health by stimulating the development of a healthy microbiota [4]. Some bacteria commonly used as probiotics for ruminants are from the genus *Bacillus*, *Bifidobacterium*, *Enterococcus*, *Lactobacillus*, *Propionibacterium*, *Megasphaera*, and *Prevotella* [5].

Recently, the use of lactic acid bacteria (LAB) as probiotics has been investigated to establish and maintain normal intestinal microbiota in ruminants with the objectives of improving food animal production and substituting antibiotics as growth promoters [6]. LAB have been shown to interact with rumen microbiota and maintain the equilibrium of the rumen ecosystem, enhance the activity of beneficial microbes, improve the degradability in the rumen, and reduce methane emissions [7, 8].

*Lactiplantibacillus plantarum* is an LAB with the potential to serve as a probiotic. Addition of *L. plantarum* cultures increased the digestion of organic matter, the production of total volatile fatty acids (VFAs) and propionic acid, and decreased the production of acetic acid and total methane. The effect of *L. plantarum* on rumen fermentation was influenced by the dosage and bacterial strain used [9, 10].

This study aimed to evaluate the effects of *L. plantarum* TSD10 as a probiotic on rumen fermentation and microbial population in Ongole breed cattle.

## Materials and Methods

### Ethical approval

The study was approved by the Animal Ethics Committee of the Indonesian Institutes of Sciences, certificate number 9879/WK/HK/XI/2015.

### Study period and location

The *in vivo* study was conducted from August to November 2016 at the Laboratory Field of Research Center for Biotechnology, National Research and Innovation Agency (BRIN) in Cibinong, West Java, Indonesia. The molecular analysis for rumen bacteria was conducted from November 2021 to February 2022 in Laboratory of Nutrition and Feed Biotechnology. The molecular analysis was done lately due to late availability of the funding.

### Experimental design

Three-year-old rumen-fistulated Ongole crossbred cattle were used in this study, arranged in a 3 × 3 crossover design. The treatments were: T0, control without probiotic; T1, with the addition of 10 mL/day probiotic *L. plantarum* TSD10; T2, with the addition of 20 mL/day probiotic; and T3, with the addition of 30 mL/day probiotic. The probiotic was given orally to the cattle at a concentration of 1.8 × 10^10^ ­colony-forming unit (CFU)/mL. *L. plantarum* TSD10 used in this study was obtained from the Biotechnology Culture Collection, Research Center for Biotechnology, National Research and Innovation Agency.

The basal diet consisted of 70% concentrate: 30% elephant grass (*Pennisetum purpureum*). The concentrate comprised 20% rice bran, 7% coffee pulp, 14% corn gluten feed, 10% coconut meal, 20% palm kernel meal, 20% pollard, 7.5% soybean meal, 0.5% mineral mix, 0.5% dicalcium phosphate, and 0.5% calcium. It contained 14% crude protein, 22.1% crude fiber, 36.4% acid detergent fiber, and 55.9% neutral detergent fiber.

Two weeks before the beginning of the experiment, cattle were fed basal diets. Each experimental period lasted 15 days and the ruminal contents were sampled thrice – on the 1^st^, 5^th^, and 10^th^ days. The ­samples were taken 3 h after morning feeding for measuring parameters through the cannula, and the ruminal pH was measured immediately. Ruminal contents were filtered using a sterilized double cheesecloth and transferred to a sterilized Corning tube. The filtrate was stored at −20°C until it was used to analyze VFAs, NH_3_, protozoa count, and microbial quantification.

### DNA extraction from rumen fluid

Microbial DNA from rumen fluid in each treatment group was extracted using the Genomic DNA Mini Kit (blood or culture cell) based on the buffy coat protocol (Geneaid Ltd., Taiwan) according to the manufacturer’s instructions. The protocol was modified to add Proteinase K (final concentration 2 mg/mL) and RNase A (final concentration 10 mg/mL), followed by incubation at 60°C for 30 min [11]. DNA was quantified on a Nanodrop Spectrophotometer (P-330, Implen Nano Photometer, Germany) by the absorbance at 260 nm. The quality of DNA was verified by gel electrophoresis of aliquots of polymerase chain reaction (PCR) product (5 μL) in 1.5% agarose and 1× TAE buffer. The extracted DNA was stored at −20°C for quantitative real-time PCR (qPCR) analysis.

### Bacterial quantification by qPCR

qPCR was performed using the Qiagen Rotor-Gene RG-6000 (Qiagen, Valencia, CA, USA). The total qPCR reaction volume of 20 μL consisted of 10 μL SYBR^®^ premix ExTaq™ (Takara, Japan), 0.4 μL each of forward and reverse primers, 7.2 μL sterile Milli-Q water, and 2 μL of the extracted DNA sample from each treatment. Species-specific PCR primers used to amplify the 16S rRNA were chosen from the literature (Table-1) [12–14]. Amplification was performed with the following cycling parameters: 95°C for 10 s, 40 cycles of 95°C for 15 s, and 60°C for 30 s [15].

**Table-1:** Primers used for quantitative real-time PCR.

Species target	Sequence	bp	Reference
*Lactiplantibacillus plantarum*	F-TCAAGCGGTGAGTGAGTTTACATT R-TCTTCGCCCCCTATTGTGGA	75	[12]
*Ruminococcus albus*	F-CCCTAAAAGCAGTCTTAGTTCG R-CCTCCTTGCGGTTAGAACA	175	[13]
*Ruminococcus flavefaciens*	F-GGACGATAATGACGGTACTT R-GCAATCYGAACTGGGACAAT	835	[14]
*Treponema bryantii*	F-AGTCGAGCGGTAAGATTG R- CAAAGCGTTTCTCTCACT	421	[14]

PCR=Polymerase chain reaction

### Parameters measurement

Rumen pH was measured with a pH meter. The concentration of NH_3_ was measured by the microdiffusion Conway method [16]. Total VFA concentration and molar proportions of VFA were analyzed using gas chromatography (GC 8A, Shimadzu Corp., Kyoto, Japan, with capillary column type containing 10% SP-1200, 1% H_3_PO_4_ on 80/100 Cromosorb WAW and nitrogen as a gas carrier). Total rumen bacteria were quantified using 9–85 medium with the rolled-tube method [17]. LAB population was quantified in terms of CFU using the Total Plate Count method. The MRS agar plates were incubated at 39°C for 24 h in anaerobic conditions, using an anaerobic jar with AnaeroPack to limit the oxygen. The number of protozoa in rumen fluid was counted under a microscope. The fresh rumen fluid (1 mL) was mixed with methyl green-formalin-salt solution (1 mL) and kept at room temperature in the dark until counting was done. Protozoa populations were counted using the Fuchs Rosenthal Counting Chamber (4 × 4 × 0.2 mm) under a microscope Olympus CX41 (Olympus, Japan) (40×) [17].

### Statistical analysis

Data were analyzed using analysis of variance with Statistical Package for the Social Sciences (IBM Corp., NY, USA) version 16 software for Microsoft Windows. Significant effects of treatments were determined by Duncan’s multiple range test. Significant differences were accepted if p < 0.05.

## Results

### Rumen fermentation

Increasing doses of probiotics resulted in different rumen fermentation products (Table-2). The control group showed the highest pH (7.05), significantly different (p < 0.05) from the treatment groups T2 (6.40) and T3 (6.14). Probiotic addition significantly increased NH_3_ production compared with control, but the effect was not dose-dependent. The effect of probiotics on VFA production followed the same trend. The control group produced the lowest VFA (108.67 mM), while the T3 group produced the highest. The proportion of acetic acid decreased with the addition of *L. plantarum*. The control group produced the highest proportion of acetic acid (64.12%), which was significantly different from the lowest proportion of acetic acid (55.53%) produced in the T3 group. On the other hand, propionic acid proportion increased with probiotic addition – the lowest was produced by the control group (18.67%), which significantly differed (p < 0.5) from that produced by the T2 (23.32%) and T3 (23.18%) groups. The proportion of butyric, isovaleric, and valeric acids was not impacted by the treatments. Probiotic treatments significantly lowered the acetate–propionate (A: P) ratio. The lowest A: P ratio resulted from the highest probiotic treatment (T3), which was significantly different (p < 0.5) from the ratio observed in the control group (3.44). A: P ratio was not affected by the dose of the probiotic.

**Table-2:** Fermentation products from* in vitro* rumen fermentation supplemented with probiotic.

Variables	Treatments			

	T_0_	T_1_	T_2_	T_3_
pH	7.05 ± 0.05^c^	6.26 ± 0.04^ab^	6.40 ± 0.13^b^	6.14 ± 0.04^a^
NH3 (mM)	13.35 ± 1.27^a^	22.08 ± 1.60^b^	20.51 ± 1.38^b^	22.40 ± 1.73^b^
Total VFA (mM)	108.67 ± 11.2^a^	160.71 ± 11.08^b^	160.18 ± 9.41^b^	178.37 ± 9.08^b^
Acetic acid (%)	64.12 ± 1.31^b^	58.77 ± 1.34^a^	57.61 ± 1.03^a^	55.53 ± 1.42^a^
Propionic acid (%)	18.67 ± 0.90^a^	21.29 ± 1.18^ab^	23.32 ± 1.00^b^	23.18 ± 1.17^b^
Isobutyric (%)	3.79 ± 0.43^b^	3.22 ± 0.14^a^	3.48 ± 0.07^ab^	3.25 ± 0.23^a^
Butyric (%)	10.85 ± 1.53	12.96 ± 0.34	0.64 ± 1.12	12.48 ± 1.22
Isovaleric (%)	2.73 ± 0.68	2.29 ± 0.17	122.14 ± 0.18	2.74 ± 0.51
Valeric (%)	1.01 ± 0.34	1.36 ± 0.27	1.15 ± 0.18	1.43 ± 0.51
A: P	3.44 ± 0.04^b^	2.77 ± 0.22^a^	2.49 ± 0.12^a^	2.41 ± 0.23^a^

T_0_=Control treatment without probiotic, T_1_=Probiotic *Lactiplantibacillus plantarum* 10 mL/day; T_2_=Probiotic *Lactiplantibacillus plantarum* 20 mL/day; T_3_=*Probiotic Lactiplantibacillus plantarum* 30 mL/day. Means in the same row with different superscripts differ significantly (p < 0.05). A: P=Acetate: propionate

### Microbial population

Probiotic treatment had no significant effect on the population of total rumen bacteria and LAB (Table-3). However, the highest dose of probiotic (T3) showed the numerically highest population of LAB (8.49 log CFU/mL). The population of protozoa, however, was significantly reduced (p < 0.05) in the rumen fluid for all doses of probiotic administered. The highest protozoan population was counted in the control group (5.83 log cells/mL), while the lowest was counted in the T2 group (5.65 log cells/mL).

**Table-3:** Population of rumen microbes from* in vitro* rumen fermentation supplemented with probiotic.

Variables	Treatments			

	T_0_	T_1_	T_2_	T_3_
Total Rumen Bacteria (log CFU/mL)	11.85 ± 0.11	11.91 ± 0.56	11.91 ± 0.26	11.57 ± 0.05
Total LAB (log CFU/mL)	8.05 ± 0.10	7.87 ± 0.58	7.53 ± 0.50	8.49 ± 0.77
Total Protozoa (log cell/mL)	5.83 ± 0.01^b^	5.69 ± 0.01^a^	5.65 ± 0.06^a^	5.73 ± 0.04^a^

LAB=Lactic acid bacteria, T_0_=Control treatment without probiotic, T_1_=Probiotic *Lactiplantibacillus plantarum* 10 mL/day, T_2_=Probiotic *Lactiplantibacillus plantarum* 20 mL/day, T_3_=Probiotic *Lactiplantibacillus plantarum* 30 mL/day. Means in the same row with different superscripts differ significantly (p < 0.05). CFU=Colony-forming unit

qPCR was used to determine the population of specific rumen bacteria: *L*. *plantarum, Ruminococcus albus*, *Ruminococcus flavefaciens*, and *Treponema bryantii* (Table-4). Probiotic treatments at any dose did not significantly affect the population of *L*. *plantarum, R. flavefaciens*, and *T. bryantii*. However, a numerical increase was observed for *L*. *Plantarum* and *R. Flavefaciens*. However, the population of *R. albus* significantly increased (p < 0.05) in the T2 and T3 groups compared with the controls. The lowest population of *R. albus* was found in the control group (9.88 log CFU/mL), while the highest was found in the T2 and T3 groups at 12.62 and 12.60 log CFU/mL, respectively. A higher dose of probiotics (T2 and T3) resulted in a numerically lower population of *T. bryantii*.

**Table-4:** Targeted bacterial population from* in vitro* rumen fermentation supplemented with probiotic.

Species	Treatments			

	T_0_	T_1_	T_2_	T_3_
*Lactiplantibacillus plantarum* (log CFU/mL)	7.88 ± 0.63	8.97 ± 0.60	7.31 ± 0.68	10.06 ± 0.59
*Ruminococcus albus* (log CFU/mL)	9.88 ± 0.16^a^	10.02 ± 0.08^a^	12.62 ± 0.18^b^	12.60 ± 0.12^b^
*Ruminococcus flavefaciens* (log CFU/mL)	9.88 ± 0.16	10.02 ± 0.08	12.62 ± 0.28	12.61 ± 0.12
*Treponema bryantii* (log CFU/mL)	7.28 ± 0.17	7.55 ± 0.15	6.79 ± 0.16	6.68 ± 0.14

T_0_=Control treatment without probiotic, T_1_=Probiotic *Lactiplantibacillus plantarum* 10 mL/day, T_2_=Probiotic *Lactiplantibacillus plantarum* 20 mL/day, T_3_=Probiotic *Lactiplantibacillus plantarum* 30 mL/day. Means in the same row with different superscripts differ significantly (p < 0.05). CFU=Colony-forming unit

## Discussion

Supplementation of the probiotic *L. plantarum* changed the fermentation products in the rumen. In this study, pH of rumen fluid decreased on adding probiotic, probably due to the lactic acid produced by this LAB. Supplementation with the probiotic Lactobacillus has been shown to lower the pH compared with the control [18]. Although the pH of the rumen fluid decreased, it was still within a normal range, with the lowest pH being 6.14 measured in the T3 group.

A significant increase in total VFA production after supplementation of *L. plantarum* is evidence that the decrease in pH did not negatively affect rumen fermentation. VFAs are metabolic products of feed digestion by rumen microbes; therefore, an increase in their production after probiotic addition indicated that the metabolic activity of rumen microbes had increased, which may help the cattle increase their productivity. This finding correlated with the significant increase in the numbers of the fibrolytic bacteria *R. albus*, which could cause VFAs to increase.

Similar to the results of the previous studies [18, 19], the proportion of VFA production in this study shifted more toward propionic acid than acetic acid, which was significantly different from the controls. Being LAB, *L. plantarum* is expected to increase lactic acid production, which may stimulate the growth of lactate-utilizing microorganisms, eventually leading to the production of propionic acid from their metabolism [18]. In this study, increased propionic acid and decreased acetic acid proportions correlated with the probiotic dose. In contrast, when *L. plantarum* was supplemented in combination with *Propionibacterium*, total VFA, and propionic acid production decreased while acetic acid production increased [15]. Supplementation of *L. plantarum* 299v did not significantly affect rumen fermentation products in calves [20]. The differences in results when *L. plantarum* or LAB were used as probiotics show that their effect depends on the type of strains, dose, and substrate [7].

The shift of rumen fermentation toward higher propionic acid and lower acetic acid production naturally resulted in a significantly decreased A: P ratio. The production of propionic acid uses H_2_ in the rumen, while the production of acetic acid releases H_2_. The decrease in A: P ratio may correlate with lower methane production from rumen fermentation [21]. When more propionic acid is produced, the supply of H_2_ for methane production is reduced. H_2_ substrate competitors will affect the growth of protozoa and methanogens in the rumen. Methane produced by the ruminant represents an energy loss for the animal that constitutes 3–10% of its gross energy intake [22] and contributes to global warming [23, 24]. Therefore, decreased methane production will increase the energy available to the animal, consequently increasing productivity. Increasing propionic acid by increasing rumen fermentation could improve growth efficiency [25]. The cumulative methane production was reduced by more than 60% on adding *L. plantarum* to an *in vitro* rumen fermentation reaction; however, the total VFA production was also significantly reduced [9].

Like the results of the previous studies, probiotic supplementation in this study did not significantly affect the population of total rumen bacteria and LAB, probably because the growth of LAB is inhibited at pH higher than 6.0 [26–28]. The protozoan count significantly decreased with the addition of probiotics. In rumen fermentation, protozoa are involved in methane production by producing H_2_, while some also produce acetic acid from their metabolism [29]. This might correlate with the decreased acetic acid production observed in this study. Protozoa have been shown to be associated with methane production [30]. Methanogenic bacteria, especially those associated with protozoa, consume H_2_ to produce methane. Therefore, a decrease in protozoa in this study might have caused methane production to drop [31]. *L. plantarum* as a probiotic could reduce methane production by reducing protozoa, thereby reducing H_2_ supply for methane synthesis. Alternatively, LAB or their ­metabolites, like bacteriocins, may either inhibit the methanogens themselves, or other rumen microbes that produce H_2_ or methyl-containing compounds, eventually inhibiting methane production [8, 32, 33].

The rumen is a complex ecosystem that houses various microbes that can degrade feed particles [34]. We estimated specific rumen bacteria from rumen fluid using qPCR. Supplementation of probiotics did not significantly increase the population of *L. plantarum*, *R. flavefaciens*, and *T. bryantii*. However, the population of *R. albus* significantly increased in the T2 and T3 groups. Numerically, fibrolytic bacteria, represented by *R. albus* and *R. flavefaciens*, were increased. Increased fibrolytic bacteria can explain the increase in total VFA, since VFA is produced from feed digestion. The increase in the *R. albus* and *R. flavefaciens* population as major cellulolytic bacteria in the rumen is attributed to the increase in the dry matter degradability, or fermentation metabolites produced by LAB. Similar findings were reported by a previous study [35]. Supplementation of probiotics in ruminants has been observed to improve fiber degradation and fermentation [36]. The role of LAB in modulating rumen microbes is still unclear. The changes in total bacteria and cellulolytic bacteria could be due to the interaction of the probiotics with the rumen microbes [37]. The adaptation of ruminal microbes to the presence of lactic acid might favor the activities of cellulolytic bacteria and increase the digestion of fibrous feeds [7]. The increase in fibrolytic bacteria also serves as evidence that supplementation of LAB as a probiotic did not negatively affect rumen fermentation.

## Conclusion

Supplementation of *L. plantarum* as a probiotic changed rumen fermentation to a higher propionic acid/lower acetic acid proportion, lowering the A: P ratio, indicating lower methane production, which provides more energy for the cattle. Based on our results, the optimum dose of *L. plantarum* as a probiotic was 20 mL/day. However, the effect of *L. plantarum* as a probiotic on feed degradation in the rumen was not evaluated in this study. Changes in rumen microbes will influence feed degradation. Therefore, the effect of *L. plantarum* as a probiotic on feed degradation should be performed in further studies.

## Authors’ Contributions

WDA: Data acquisition and drafted and revised the manuscript. RR: Supervised the study, data acquisition, and revised the manuscript. RF: Analyzed data. RoR: Probiotic preparation. NFS: q-PCR analysis. KAS, AF, and YW: Revised the manuscript. All authors have read and approved the final manuscript.
